# Elevated Levels of Serum IL-17A in Community-Dwelling Women with Higher Depressive Symptoms

**DOI:** 10.3390/bs8110102

**Published:** 2018-11-04

**Authors:** Hirohito Tsuboi, Hiroyuki Sakakibara, Yuuki Minamida, Hiromasa Tsujiguchi, Masahiro Matsunaga, Akinori Hara, Hiroyuki Nakamura

**Affiliations:** 1 Institute of Medical, Pharmaceutical & Health Sciences, Kanazawa University, Kanazawa 920-1192, Japan; minamida.yuuki@stu.kanazawa-u.ac.jp; 2 Department of Biochemistry and Applied biosciences, Faculty of Agriculture, University of Miyazaki, Miyazaki 889-2192, Japan; hiroyuki@cc.miyazaki-u.ac.jp; 3 Department of Public Health, Graduate School of Medicine, Kanazawa University, Kanazawa 920-8640, Japan; t-hiromasa@med.kanazawa-u.ac.jp (H.T.); ahara@m-kanazawa.jp (A.H.); hnakamu@staff.kanazawa-u.ac.jp (H.N.); 4 Department of Health and Psychosocial Medicine, Aichi Medical University School of Medicine, Nagakute 480-1195, Japan; matsunag@aichi-med-u.ac.jp

**Keywords:** depressive symptoms, interleukin-17, community-dwelling women

## Abstract

Recent studies indicate that patients with depression have increased concentrations of serum pro-inflammatory cytokines. However, studies of IL-17 and studies on community-dwellers are few. The purpose of this study was to investigate serum cytokine levels, especially IL-17A, among subjects with high and low depressive symptoms of a general population. The participants comprised 20 female community-dwellers aged 40 years or older who contributed to a Shika study in Ishikawa, Japan. Ten participants who showed higher and ten who showed lower depressive symptoms among 208 females assessed by the Japanese version of the Centre for Epidemiologic Studies Depression Scale (CES-D) were selected for this study. Serum samples were analyzed for TNF-alpha, IL-6, IL-10, IL-12, and IL-17A using a multiplex Luminex analysis. For the comparison between the high and low depressive groups statistically, linear regression analyses were applied. The serum level of IL-17A was significantly higher among the high depressive participants (*p* < 0.05) even after controlling possible confounders, whereas there were no differences in TNF-alpha, IL-6, IL-10, or IL-12 between the high and low depressive groups. Our findings supported an association between serum IL-17A levels and depressive symptoms. Peripheral IL-17A immune response may be a preventive and treatment target for depression.

## 1. Introduction

Chronic low-grade inflammation has been examined in relation to clinical depression and/or depressive symptoms [1,2]. Chronic inflammation differs from normal or acute inflammation in that the body is unable to suppress the immune response, which results in continuous systemic low-grade inflammation. The link between inflammatory indicators and psychological or psychiatric conditions has been reported in clinical depression and depressive symptoms of healthy subjects. A meta-analysis on major depression concluded that two pro-inflammatory cytokines, interleukin (IL)-6 and tumor necrosis factor-alpha (TNF-α), were consistently elevated in depression, while other cytokines IL-1β, IL-4, IL-2, IL-8, IL-10, and interferon-γ were not consistently elevated in depressed patients [3]. Other meta-analyses have suggested associations between depression and inflammatory markers, such as C-reactive protein (CRP), IL-6 and, to a lesser extent, IL-1 [4,5]. Several pro-inflammatory cytokines play crucial roles in inflammatory reactions associated with depression [6]. Circulating peripheral cytokines lead to central inflammatory cytokines secreted by brain microglia, which plays a crucial role in regulating depressive symptoms [7]. 

IL-17A is a cytokine that plays a key role in immune activation and is associated with chronic inflammatory conditions. It is secreted mainly by T-helper 17 (Th17) lymphocytes but also by other T cells, granulocytes, monocytes, and natural killer cells, etc. The role of Th17 cells in depression has recently been investigated and there is growing evidence that inflammation promotes depression. It was hypothesized that peripheral IL-17 levels would be elevated in the peripheral blood of depressed patients. Indeed, one study found elevated serum levels of IL-17A in the blood of depressed patients [8]. Another study on 41 patients with major depressive disorder (MDD) indicated higher concentrations of serum IL-17A in comparison with healthy controls [9]. Studies about anti-depressant-SSRI treatment of depression revealed decreased peripheral IL-17 levels by some SSRIs [10,11]. Two studies with animal models have also demonstrated that increased levels of IL-17A are associated with depression-like behaviors [12,13]. It may be hypothesized that peripheral IL-17 levels would be elevated in depressed persons.

Despite the growing reports of associations between IL-17 and depression, studies on the general population are very sparse. Therefore, we investigated serum IL-17A concentrations in high and low depressive subjects in addition to tumor necrosis factor (TNF)-α, IL-6, IL-12 (pro-inflammatory cytokines), IL-10 (an anti-inflammatory cytokine), and high sensitivity C-reactive protein (hs-CRP). TNF-α is an adipose tissue-derived cytokine, which accelerates low-grade inflammation by constituting a vicious cycle between adipocytes and macrophages [14]. IL-6 has often been reported to be elevated in depressive patients [4], and IL-12 acts on Th1-polarizing [15]. IL-10 made by Th2 cells lowers IL-12 production and thereby suppresses Th1 differentiation [16]. The aim of this study was to investigate cytokine levels, especially IL-17A ones, between subjects with high and low depressive symptoms.

## 2. Materials and Methods

### 2.1. Study Population

Subjects were selected from participants in the “Shika study” project of 2015–2016, which has been carried out in Noto Peninsula, Ishikawa, Japan since 2011. This project aims to identify solutions developed for lifestyle diseases by investigating community-dwelling people aged 40 years or older. Details of this study have been previously reported elsewhere [17]. All the respondents were literate, understood the Japanese language well, and were requested not to use proxy respondents. The study protocol was approved by the Ethical Committee at Kanazawa University (on 18 December 2013, receipt number 1491). Written informed consent was obtained in all cases.

### 2.2. Procedures

A self-administrated questionnaire was distributed to the participants beforehand and collected on the examination day. The entire process was conducted with attention to the protection of the attendees’ privacy.

The questionnaire contained demographic measures (age, sex, height, weight, present health status, diseases, medication, etc.) and lifestyle characteristics (smoking status, alcohol consumption, and leisure-time physical activities). Depressive symptoms were assessed using the Japanese version of the Centre for Epidemiologic Studies Depression Scale (CES-D) [18,19]. In total, 409 (200 males: mean age 59.1 (S.D. 11.5), 208 females: mean age 61.1 (S.D. 12.9), 1 unknown) participants among 492 gave their consent to participate in this study.

**Figure 1:**
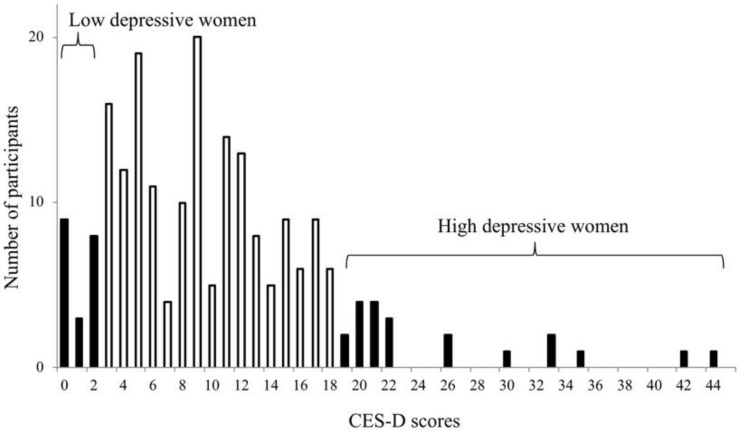
Distribution of the Centre for Epidemiologic Studies Depression Scale (CES-D) scores among 208 female participants. Ten women with the highest depressive symptoms and ten women with the lowest depressive symptoms were selected for the present study.

In this study, ten women with the highest depressive symptoms (CESD scores 19–40) and ten women with the lowest depressive symptoms (CESD scores 0–2) were selected for inflammatory marker detection (Figure 1). The demographics are presented in Table 1.

### 2.3. Blood Collection

Fasting blood was sampled between 0800 and 1200 from the forearm vein of each participant with heparinized and serum-separator vacutainer tubes, from which sera were obtained using centrifugation. The serum samples were delivered to Kanazawa University through a commercial laboratory (SRL Kanazawa Laboratory, Kanazawa, Japan). The sera were frozen and stored at −30 °C until the assay.

### 2.4. Inflammatory Assays

The serum samples were analyzed for TNF-α, IL-6, IL-10, IL-12p70, and IL-17A, using a multiplex human immunoassay kit (Luminex^®^ 200™) with the human high sensitivity T cell panel (Merck Japan, Tokyo, Japan). The hs-CRP of the serum was detected at the SRL Kanazawa Laboratory.

### 2.5. Statistical Analysis

To analyze the data, the Japanese version of the IBM SPSS Statistics 24 was used (IBM Japan, Tokyo, Japan). The high and low depressive groups were compared using basic data, lifestyles, and serum indicators utilizing an unpaired t test, and a liner regression analysis which was applied to adjust possible confounders. IL-6, IL-10, IL-12, IL-17A, and hs-CRP were log 10 transformed for the analysis. Chi-square and Fisher’s exact tests were applied for the comparison of the qualitative values. The significance is reported at *p* < 0.05.

## 3. Results

Figure 1 indicates the female participants selected. Table 1 presents the comparison of the high and low depressive participants. All the participants lived independently, and none used nursing services.

**Figure 2:**
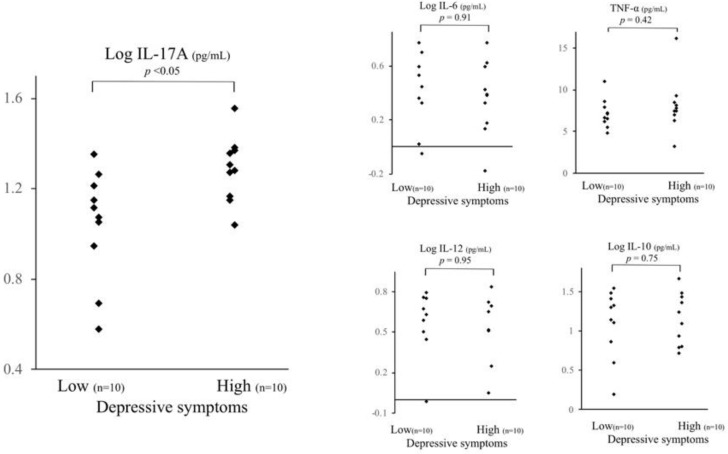
Comparison of serum cytokine levels between high and low depressive participants. The IL-17A serum level of high depressive participants was significantly higher than that of low depressive participants (*t* test: *p* < 0.05, analysis of covariance: *p* < 0.05). There were no significant differences in other serum cytokine levels between the high and low depressive participants in the other serum cytokine levels, even adjusting for age and body mass index. Depressive symptoms were assessed using the Japanese version of the Centre for Epidemiologic Studies Depression Scale. * < 0.05, n.s. not significant.

As shown in Table 1 and Figure 2, the IL-17A serum level of the high depressive participants was significantly higher than that of the low depressive participants (*t* test: *p* < 0.05). The significance did not change, even after controlling for age, body mass index (BMI), smoking habit, alcohol consumption, educational years, and living conditions (*p* < 0.05). In contrast, there were no significant differences in the serum concentrations of TNF-α, IL-6, IL-10, or IL-12 between the high and low depressive participants (Table 1, Figure 2). Hs-CRP did not differ between the two groups. Moreover, there were no significant differences in smoking habit, alcohol consumption, marital status, or living alone between the high and low depressive participants (Chi-square test). Years of education did not differ between the two groups (*t* test). 

## 4. Discussion

The current investigation compared the peripheral cytokine levels of high and low depressive community-dwelling female participants. We identified significant elevated serum levels of IL-17A among high depressive participants in comparison with low depressive participants, even controlling for possible confounders. In contrast, TNF-α, IL-6, IL-10, IL-12, and hs-CRP did not exhibit any significant differences between the two groups. 

### 4.1. IL-17 and Depression

Serum IL-17A levels in the higher depressive participants were significantly higher compared to those of the lower depressive participants. The participant who had the highest depressive status took an anti-depressant (trazodone), anxiolytic (alprazolam, diazepam), and sleeping medicine (triazolam). Taking into consideration the inflammatory effect of these medicines on IL-17, it appears there was no problem with including this participant in the analyses. Because no report concerning the relationship between the above-mentioned medicines and IL-17 was found [20,21,22,23], and because alprazolam and diazepam generally have a controversial immune response [15], simultaneous administration of them may have no effect on immune functions. In addition, this participant had a habit of leisure-time physical activities among the higher depressive group (Table 1), suggesting that the depressive status of the patient might not be so high as to affect their behavior or physical conditions, including their immune responses. Furthermore, anti-depressant treatment of depression could decrease peripheral IL-17A levels. Escitalopram and sertraline were found to decrease plasma IL-17 levels in MDD patients [11]. Higher baseline levels of IL-17 are selectively associated with greater symptomatic reduction in MDD patients treated with a bupropion combination [10]. In an animal experiment on C57BL/6 male mice using a zoosocial stress model and evaluating depression-like behavior using an actometer and the elevated plus-maze, the serum concentration of IL-17 was found to increase with stress and decrease after anti-depressant treatments with ladasten and imipramine [13].

### 4.2. IL-6 and Depression

The inflammatory cytokines in the central nervous system, specially IL-6, can lead to depression through neuroinflammation [4,24]. However, considering a systematic review on IL-6 and depression, the number of participants in the present study appeared to be too small to examine the IL-6 differences between the two groups [25,26]. In another study of ours (data not shown), serum IL-6 levels and depressive symptoms were barely correlated (*p* < 0.05) among 133 female subjects. IL-17A may be more sensitive than IL-6 in relation to depressive status.

### 4.3. TNF-α, IL-10, IL-12 and Depression

Compared to IL-6, reports indicated that the relationships between depression and TNF-α, IL-12 and IL-10 are few [3,4]. These cytokines appear not to be related with depression differently from IL17A and IL-6, though we had predicted a reduction in anti-inflammatory cytokine IL-10 in the high depressive participants, as shown in an animal experiment: social stress on C57Bl/6J mice induces depression-like behavior, concomitant with the hypoproduction of IL-10 [27]. Rather, the IL-10 levels were significantly positively correlated with pro-inflammatory cytokines IL-6, IL-12, and IL-17A (simple correlation: *p* < 0.0005, *p* < 0.0005, *p* < 0.05, respectively) in the present study.

### 4.4. Limitations

Because this was a cross-sectional study, causal associations cannot be made. Secondly, the number of participants was small, which might cause type I error or unknown confounders affecting the results. Thirdly, the use of self-reporting to assess depressive symptoms is unable to make an accurate diagnosis of MDD.

## 5. Conclusions

In summary, we found elevated IL-17A serum levels among high depressive female community-dwellers. To our knowledge, this is the first report indicating the elevation of serum IL-17A levels in a community-dwelling population. This result may suggest a pathogenic role of IL-17A in depression. Furthermore, it can be applied by education and policy-makers in relation to anti-inflammatory lifestyles to prevent depression.

## Figures and Tables

**Table 1:** Comparison between high and low depressive symptoms.

Variable	High Depressive (n = 10)	Low Depressive (n = 10)	*p* Value
Age	59.7 (15.53)	60.6 (12.8)	0.87
BMI	21.5 (3.69)	23.0 (3.67)	0.37
CES-D scores	24.4 (8.1)	0.8 (1.03)	<0.0005
IL-6 ^a^ (pg/mL)	2.71 (1.555)	2.8 (1.789)	0.91
IL-10 ^a^ (pg/mL)	18.2 (13.27)	17.1 (11.15)	0.75
IL-12 ^a^ (pg/mL)	5.06 (2.982)	4.74 (2.362)	0.95
IL-17a ^a^ (pg/mL)	20.3 (6.93)	12.5 (5.75)	0.01
TNF-α (pg/mL)	8.19 (3.269)	7.22 (1.748)	0.42
hs-CRP ^a^ (mg/dL)	0.12 (0.238)	0.06 (0.050)	0.99
Smoking habit			
no smoker	9	9	0.37 ^c^
ex-smoker	0	1	
Present smoker	1	0	
Alcohol consumption			
No habit	7	8	0.79 ^c^
Several days per week	3	0	
Everyday	0	2	
Leisure-time physical activities ^b^	2	1	0.50 ^d^
Educational years	11.5 (2.92)	10.4 (1.51)	0.31 ^c^
Marital status			
Married	6	6	0.37 ^c^
Single	3	1	
Widowed (more than three years before)	1	3	
Living alone	2	1	0.50 ^d^

Note: Values were expressed mean (S.D.), *p* values due to *t* test. n.s. not significant. BMI: body mass index, CES-D: the Centre for Epidemiologic Studies Depression Scale, hs-CRP: high sensitivity C-reactive protein, IL: interleukin, TNF: tumor necrosis factor. ^a^ Analises were carried out after log10 transformed. ^b^ Over 30 minutes exercise more than two days every week was continued over one year. *p* values due to *t* test. ^c^ chi-suare test. ^d^ Fisher’s exact test.
